# Report of a Rare Case of Pubic Symphysis Tuberculosis in an Eight-Year-Old Child: An Unusual Presentation of the Disease

**DOI:** 10.7759/cureus.36149

**Published:** 2023-03-14

**Authors:** Roop Singh, Isha Seth, Ram K Aiyappan, Sunayana Singh, Aditya Seth, Anish Tawde, Chander M Yadav, Harsh Jain

**Affiliations:** 1 Orthopedics, Pandit Bhagwat Dayal Sharma Post Graduate Institute of Medical Sciences, Rohtak, IND; 2 Obstetrics and Gynecology, Amrita Hospital, Faridabad, IND; 3 General Surgery, Amrita Hospital, Faridabad, IND; 4 Obstetrics and Gynecology, Pandit Bhagwat Dayal Sharma Post Graduate Institute of Medical Sciences, Rohtak, IND; 5 Arthroplasty, Krishna Institute of Medical Sciences, Sunshine Hospital, Hyderabad, IND; 6 Orthopedics and Rehabilitation, Pandit Bhagwat Dayal Sharma Post Graduate Institute of Medical Sciences, Rohtak, IND

**Keywords:** osteitis pubis, osteomyelitis, extra-pulmary tuberculosis, pubic symphysis, tuberculosis

## Abstract

Tuberculosis is a well-known and ancient disease with a wide range of clinical presentations. Although tuberculosis is a well-known infectious disease, involvement of the symphysis pubis is rare, with only a few documented cases in the medical literature. Distinguishing it from other more common conditions, such as osteomyelitis of the pubic symphysis and osteitis pubis, is essential to avoid delay in diagnosis and to minimize morbidity, mortality, and complications. We present a rare case of tuberculosis of the symphysis pubis in an eight-year-old female from India who was initially misdiagnosed with osteomyelitis. Following the correct diagnosis and commencement of anti-tuberculosis chemotherapy, the patient demonstrated improvement in symptoms and hematological parameters at the three-month follow-up. This case emphasizes the importance of considering tuberculosis as a differential diagnosis in cases of symphysis pubis involvement, especially in areas with a high incidence of tuberculosis. Early diagnosis and appropriate treatment can prevent further complications and improve clinical outcomes.

## Introduction

Osteoarticular tuberculosis is responsible for approximately 15% of all extrapulmonary tuberculosis cases. Only nine occurrences of tuberculosis of the pubis symphysis have been recorded in the last three decades [1]. However, in the pre-chemotherapy era, up to 100 cases were reported in the early 20th century, all of which were diagnosed in advanced stages. The first case of pubic symphysis tuberculosis in the literature was published by Jackson in 1923 [2].

Symphysis pubis inflammation can be either infectious or non-infectious in origin, with osteitis pubis being a self-limiting non-infectious inflammation caused by trauma, pelvic surgery, delivery, or overuse, while pubic symphysis osteomyelitis is largely bacterial in etiology and associated with pelvic malignancies, urological, trauma, and gynecological treatments, as well as intravenous drug abuse [3-7].

Despite the decline in tuberculosis cases and improved treatment success rates, tuberculosis remains a significant global health concern, particularly in the context of high co-infection rates in HIV-positive individuals and the emergence of multidrug-resistant *Mycobacterium tuberculosis* cell lines [8]. Due to its rarity, elusive detection, and the need for prolonged antibiotic therapy, musculoskeletal tuberculosis presents a considerable challenge, frequently resulting in treatment failures [9].

Following lymph nodes, osteoarticular tuberculosis is the second most common extrapulmonary tuberculosis presentation, accounting for approximately 13% of cases [3,10]. The most commonly affected skeletal sites are the spine, followed by the hip, knee, and ankle joints. Any bone or joint in the body can be affected by tuberculosis. The infection causes bone destruction and the formation of a metaphyseal abscess, which leads to the loss of nutrients to the hyaline cartilage. Localized suppurative reactions may directly or indirectly degrade the cartilage [11]. The pubic symphysis and its ligaments act as a tension band for the rotational stresses applied to the pelvis in the upright posture. In the advanced stages of the disease, the loss of the symphysis and pubic bones may result in secondary sacroiliac strain, symphysis diastasis, and pelvic instability if left untreated. Tuberculosis of the pelvic girdle is primarily localized to the sacroiliac joint, with localized inclusion of the ilium or ischial tubercle occurring less frequently [12].

## Case presentation

An eight-year-old female patient presented to Pandit Bhagwat Dayal Sharma Post Graduate Institute of Medical Sciences (PGIMS) Rohtak, Haryana, India, with a complaint of suprapubic pain and soft tissue edema persisting for four weeks. The pain worsened upon standing or walking, while activities such as coughing, sneezing, voiding, or straining did not aggravate the symptoms. The patient had no history of trauma, athletic effort, infection, or surgery. She reported a low-grade evening rise in temperature and nocturnal sweats but no weight loss or coughing in the past months. On inspection, the pubic symphysis was tender, but there was no inguinal lymphadenopathy or abnormality during the rectal exam.

**Figure 1:**
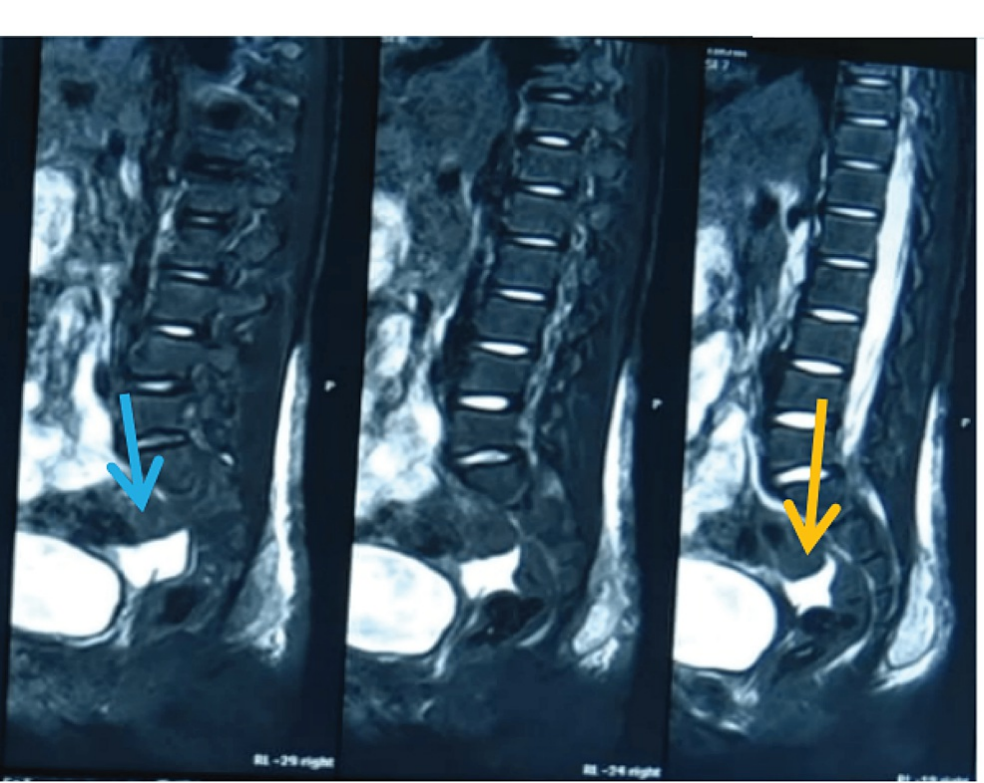
Initial MRI (done one month prior to presentation to our center) revealing evidence of free fluid and collection anterior to sacrum

The patient had previously visited a private hospital where an MRI scan of the lumbosacral spine revealed evidence of destruction with marrow edema involving the left pubic bone with irregular multi-loculated altered signal intensity collection along the left pelvic wall, left gluteal region and anterior to sacrum predominantly in intra/intermuscular plane. The collection measures 62x84x105mm (APxTRxCC) involving the left gluteus maximus and left obturator internus muscle extending to the left ischio-anal fossa. Mild free fluid was also seen in the pelvis (Figure 1).

**Figure 2:**
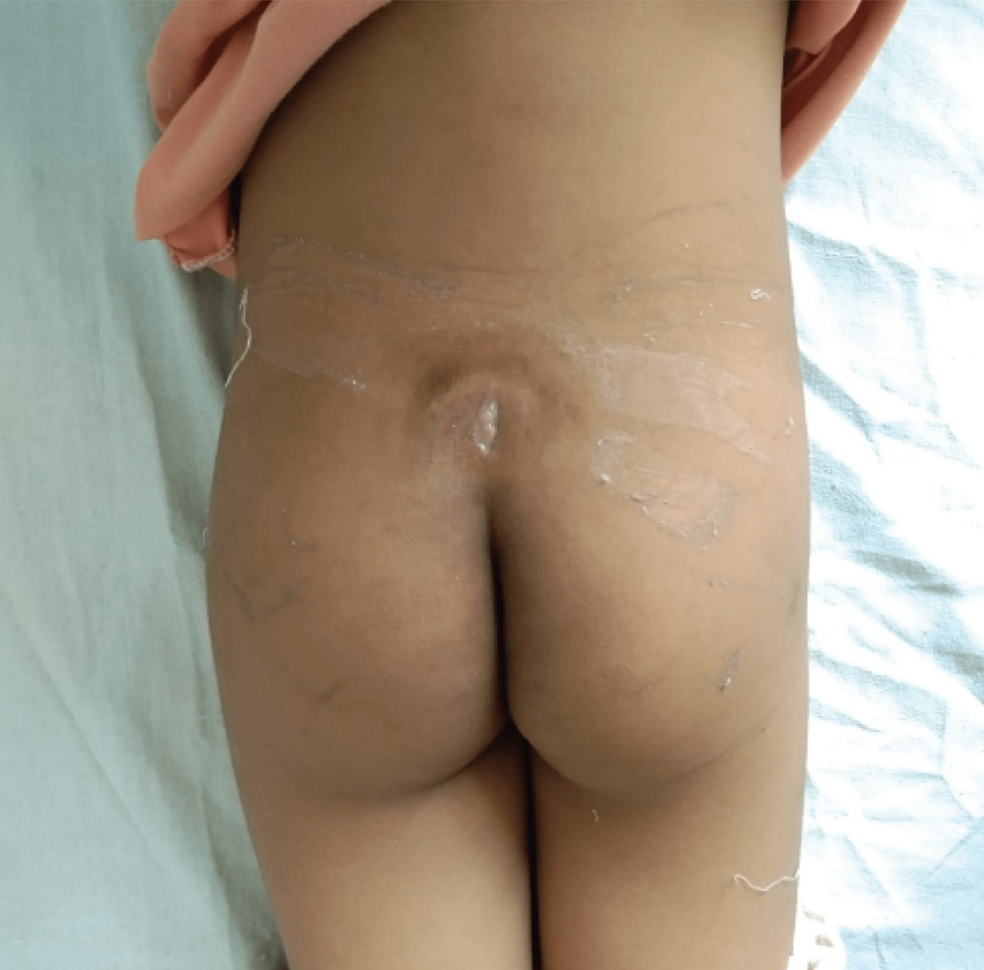
Site of incision given in a private hospital one month before when the patient reported at our center

Based on these MRI findings, a small vertical incision was given above the gluteal cleft, and pus was drained (Figure 2); however, no pus culture was sent. A diagnosis of osteomyelitis of pubic bone was made, and empirical antibiotics were given to the patient for one month. However, when the patient's symptoms didn't improve, she reported to our center.

**Figure 3:**
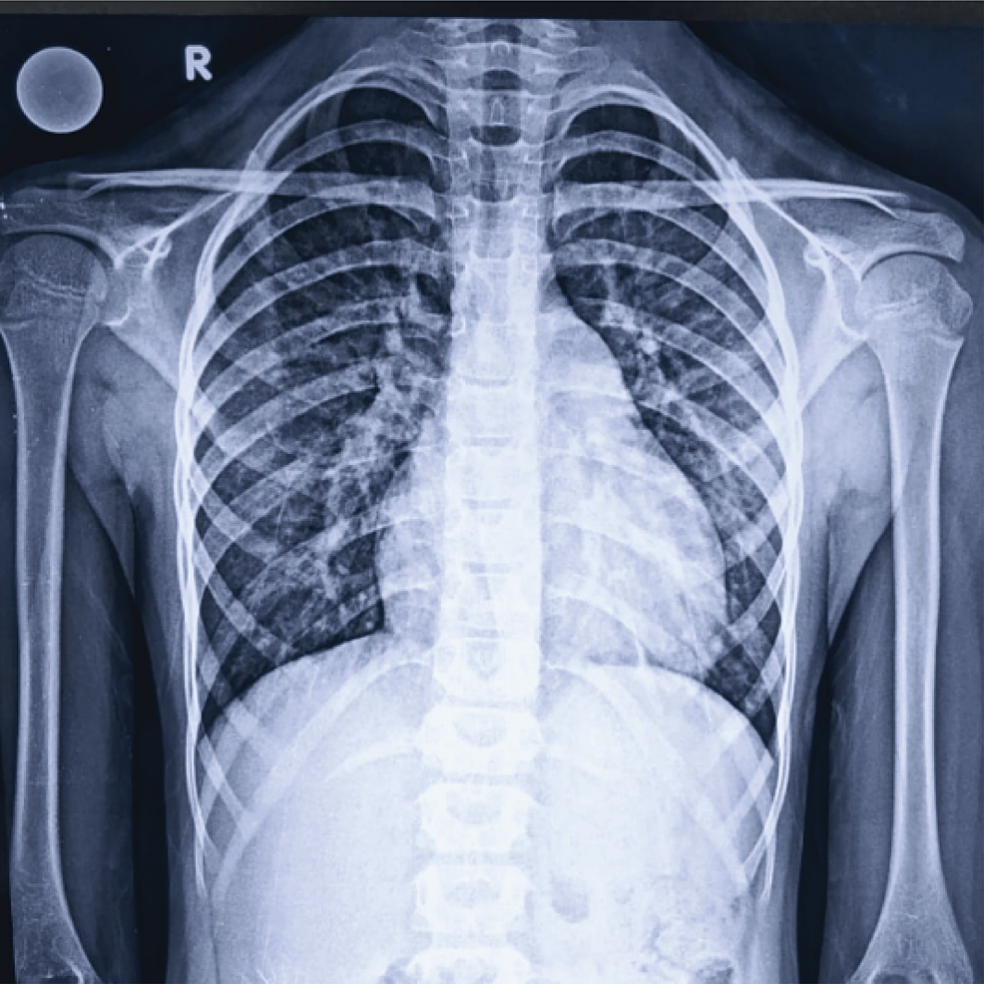
Chest radiograph revealing no abnormality

**Figure 4:**
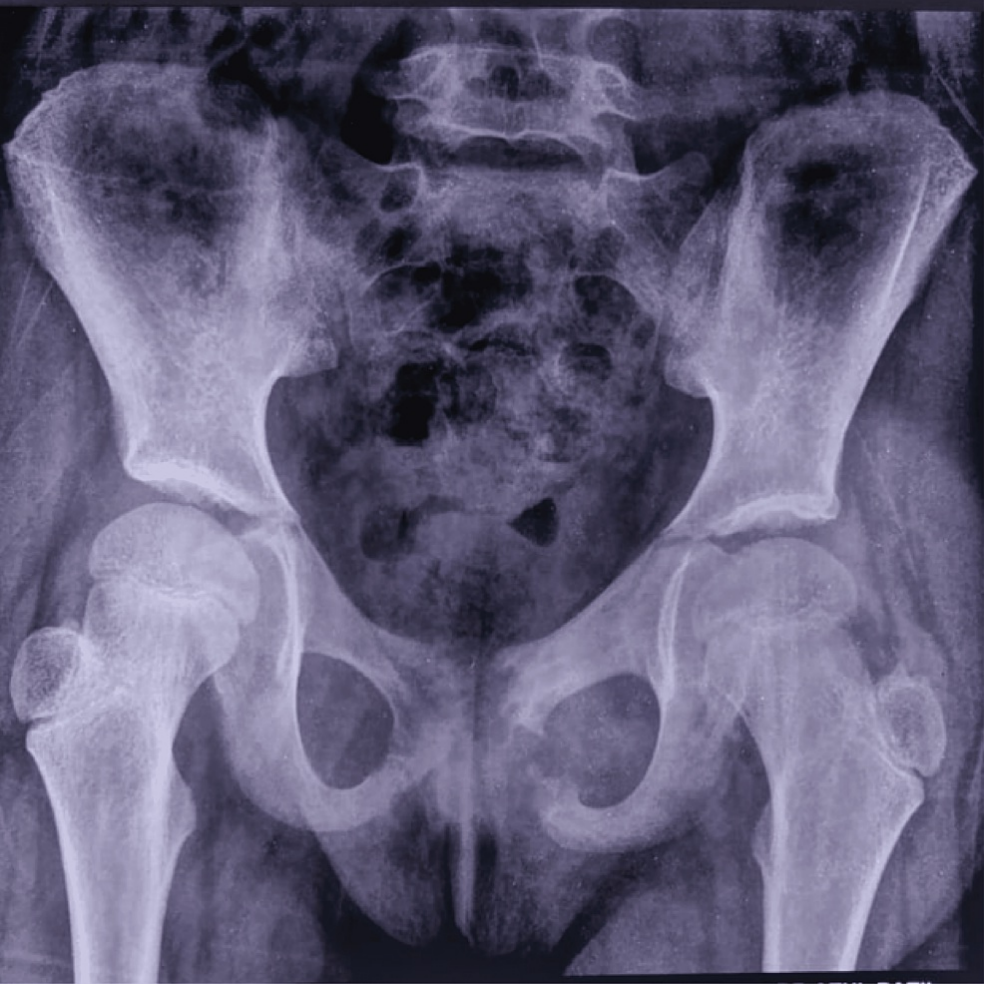
X-Ray of pelvis revealing bony erosion of pubic symphysis

Laboratory tests revealed an elevated total leukocyte count (TLC), erythrocyte sedimentation rate (ESR), and C-reactive protein (CRP) (Table 1). Chest radiographs were normal (Figure 3), but X-rays of the pelvis revealed bony erosion of the pubic symphysis and pubic bone (Figure 4).

**Table 1:** TLC, ESR, and CRP values at presentation, at three months, and at six months TLC - total leukocyte count, ESR - erythrocyte sedimentation rate, CRP - C-reactive protein

Test	Reference range	Value at presentation to our center	Value at 3 months of treatment	Value at 6 months of treatment
Total leukocyte count	4000-11000/mm^3^	13,400 / mm^3^	8200/mm^3^	7600/mm^3^
Erythrocyte sedimentation rate	0-20mm/hr	45 mm/hr	26 mm/hr	16mm/hr
C-reactive protein	0-6mg/l	45 mg/l	8 mg/l	4 mg/l

**Figure 5:**
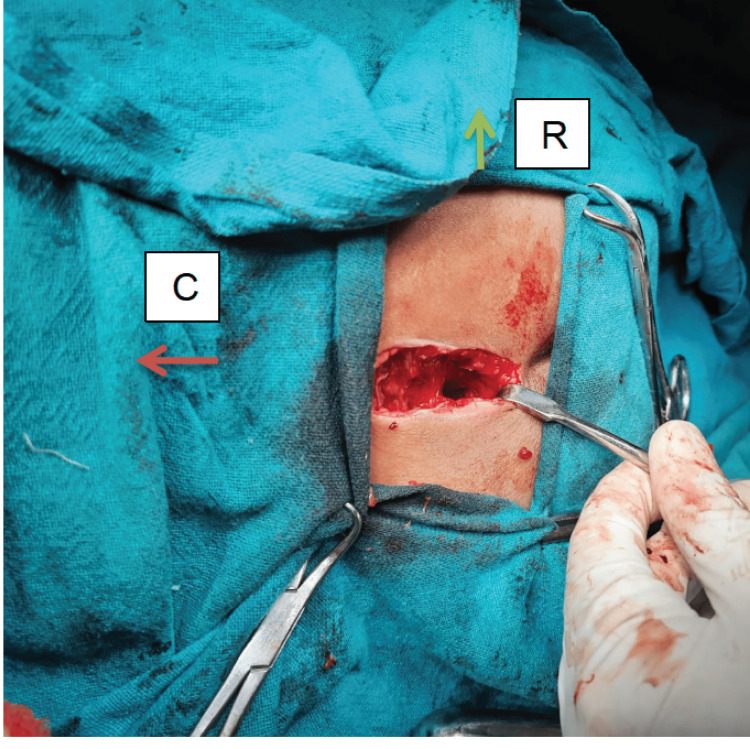
Site of biopsy C - cranial, R - right

Fine needle aspiration cytology (FNAC) was initially attempted but was unsuccessful, and the patient underwent a biopsy under general anesthesia. The biopsy (Figure 5) revealed caseous necrosis and epithelioid cell clusters intermingled with histiocytes. The sample was also sent for a cartridge based nucleic acid amplification test (CBNAAT), which was positive for tuberculosis bacilli without rifampicin resistance. Thus, the patient was diagnosed with tuberculosis of the symphysis pubis and started on a multidrug antitubercular treatment regimen that included rifampicin, isoniazid, ethambutol, and pyrazinamide. After three months of medication, the patient's symptoms improved, and her blood parameters normalized. These were further improved when the patient was telephonically consulted at six months of follow-up and was advised for a blood test (Table 1).

## Discussion

Tuberculosis of the pubis commonly presents with infection and swelling in the perineal, hypogastric, medial thigh, or ischiorectal area, which can initially be asymptomatic. Fatigue in the legs and a limp when awakened are typically the first signs, often followed by pain in the hip with slightly reduced abduction. Patients typically seek medical attention when they develop an abscess (as in our case) in the community of the symphysis, medial side of the thigh, or anal area [13]. These abscesses can lead to sinus or fistula formation, cold abscess, or hypogastric mass, causing significant morbidity and mortality [2].

Differential diagnoses in such cases may include osteomyelitis, osteitis pubis, and adolescent osteochondritis of the symphysis pubis [14]. Osteitis pubis is a non-infectious, self-limiting inflammation of the pubic symphysis commonly seen in athletes during pregnancy, after gynecological and urological surgeries, or after trauma to the pubic symphysis [2]. Unlike tuberculosis of the pubis, osteitis pubis is characterized by severe discomfort over the pubic symphysis without any evidence of abscess formation. Initial radiographs may reveal patchy sclerosis, uneven cortical borders, and significant pubis rarefaction, but a sequestrum is rare. Treatment of osteitis pubis includes moist heat, rest, and nonsteroidal anti-inflammatory medicines. 

Pyogenic osteomyelitis of the pubic symphysis can mimic the symptoms of gynecological and urological procedures. Pyogenic infection of the pubis, rather than TB of the symphysis pubis, may be a more typical occurrence. Microorganisms isolated from the lesion are used to confirm the diagnosis. Gulia et al. described a case of pubic symphysis tuberculosis with a draining inguinal fistula that was successfully treated with antitubercular chemotherapy without the need for surgery [15]. The isolation of the organism determines the condition's diagnosis. The most common organism isolated is *Staphylococcus aureus*, followed by *Pseudomonas*. Even in cases where antibiotics were given, Knoeller et al. proved that the bacterium could be cultivated [16].

Magnetic resonance imaging (MRI) can identify an abscess in the perineal, medial thigh, hypogastric, or ischiorectal area, and polymerase chain reaction (PCR) and culture can confirm the diagnosis by isolating acid-fast bacilli from pubic foci-aspirate. The presence of elevated fluid adenosine deaminase levels may aid in the diagnosis of tuberculosis. In cases where surgery is required, it is typically to discharge large abscesses or retrieve tissue samples, and bone grafting should be avoided on an infected bed [2].

## Conclusions

In conclusion, tuberculosis of the pubis is a serious condition that can cause significant morbidity and mortality if left untreated. Early diagnosis and prompt treatment with antitubercular chemotherapy or antibiotics can be effective in many cases, but surgical intervention may be necessary in some cases, particularly when complications arise. Clinicians should be aware of the differential diagnoses, such as osteitis pubis and pyogenic osteomyelitis, and consider performing PCR, culture, or needle cytology to confirm the diagnosis.
